# Photothermal hydrogels for infection control and tissue regeneration

**DOI:** 10.3389/fbioe.2024.1389327

**Published:** 2024-03-28

**Authors:** Siyu Sun, Guangyang Jiang, Jianru Dong, Xi Xie, Jinfeng Liao, Yongqiang Tian

**Affiliations:** ^1^ College of Biomass Science and Engineering, Sichuan University, Chengdu, China; ^2^ State Key Laboratory of Oral Diseases, National Clinical Research Centre for Oral Diseases, West China Hospital of Stomatology, Sichuan University, Chengdu, China

**Keywords:** photothermal hydrogels, infection control, tissue regeneration, near-infrared stimulation, photothermal agents, biomedical materials

## Abstract

In this review, we report investigating photothermal hydrogels, innovative biomedical materials designed for infection control and tissue regeneration. These hydrogels exhibit responsiveness to near-infrared (NIR) stimulation, altering their structure and properties, which is pivotal for medical applications. Photothermal hydrogels have emerged as a significant advancement in medical materials, harnessing photothermal agents (PTAs) to respond to NIR light. This responsiveness is crucial for controlling infections and promoting tissue healing. We discuss three construction methods for preparing photothermal hydrogels, emphasizing their design and synthesis, which incorporate PTAs to achieve the desired photothermal effects. The application of these hydrogels demonstrates enhanced infection control and tissue regeneration, supported by their unique photothermal properties. Although research progress in photothermal hydrogels is promising, challenges remain. We address these issues and explore future directions to enhance their therapeutic potential.

## 1 Introduction

Tissue defects are a common health problem involving the damage or loss of internal tissues in organisms. Such defects can be caused not only by external trauma but also by factors such as disease and surgery, or they may be congenital. Tissue damage covers a complex and wide range from skin surface damage to the loss of deep organs. For example, wound defects may be caused by trauma (Pan et al., 2022), burns (Contardi et al., 2021), or cuts (Tang et al., 2021), whereas fractures are structural damage to bone caused by trauma (Zhang et al., 2023). In addition, tissue defects increase the risk of infection (Qian et al., 2023) because the normal physiological barriers are destroyed when tissue is damaged, enabling the entry of bacteria, viruses, and other pathogens to trigger infections. Owing to the limited regenerative capacity of numerous tissues after external injury, current clinical approaches rely heavily on surgical repair and organ transplantation (Liu et al., 2021). However, these therapeutic approaches face numerous challenges, including the risk of immune rejection, increased risk of secondary infection, and the need for the functional recovery of damaged tissues. Given these challenges, there is an urgent need to explore novel, safe, and efficient treatments.

There have been continuous advancements in the field of biomaterials, and the development of those having tissue regeneration capabilities has become a research hotspot (Chen and Liu, 2016). Various biomaterials, including films (Savencu et al., 2021), hydrogels (Cai et al., 2022), cotton (Guo et al., 2022), sponges (Shakiba-Marani and Ehtesabi, 2023), and three-dimensional (3D) printing scaffolds (Wang et al., 2019), have been extensively studied. Recently, hydrogels have garnered significant attention because of their remarkable properties. As multifunctional polymer materials, hydrogels have excellent biocompatibility, outstanding water absorption, and extracellular matrix (ECM)-like 3D porous structures (Liang et al., 2021). Crucially, the biocompatibility of hydrogels reduces the likelihood of immune response or tissue rejection, and their remarkable water absorption capacity enables them to absorb large amounts of water while maintaining a stable gel state, facilitating tight contact with wounds without adhesion, thus reducing bacterial contact. Further, the 3D network structure of hydrogels mimics that of the ECM, providing a conducive environment for cell growth and tissue regeneration (Malik et al., 2023). Researchers have also introduced injectable hydrogels to conveniently address the healing of irregularly damaged tissues (Li et al., 2021). Moreover, the properties of hydrogels can be finely tuned during tissue healing by manipulating their composition. This involves integrating drugs, growth factors, and other biologically active substances to provide a controlled environment for enhanced tissue repair (Hu et al., 2021). Considering these characteristics, hydrogels have significant potential for tissue repair and regeneration. In addition, to improve the therapeutic effect, the antibacterial properties of hydrogel materials are particularly important (Zhang et al., 2022).

The infection of damaged tissues has long been a challenge in the medical field (Bielka et al., 2023). Infection with bacteria, viruses, or other pathogenic microorganisms hinders natural healing and prolongs recovery time (Wang et al., 2021). Although antibiotics play an important role in clinical practice, their application has resulted in several issues, such as bacterial resistance and allergic reactions (Jourdan et al., 2020; Li et al., 2021; Huang et al., 2022; Duan et al., 2022). To overcome these problems, researchers have focused on temperature regulation in bacterial, cellular, and tissue responses (Xu et al., 2022). Photothermal therapy (PTT) is an innovative and efficient treatment. By inducing a thermal effect under specific light wavelengths, PTT offers a solution for the treatment of bacterial infections (Chen et al., 2020). This therapy relies on specific photothermal agents (PTAs) that generate thermal effects during treatment. PTAs include diverse materials, such as metal nanoparticles (NPs), carbon-based nanomaterials, and organic dyes (Li et al., 2021; Yang et al., 2021; Lagos et al., 2022). Each class has unique optical and thermal attributes, making the selection of an appropriate PTA critical for designing an effective PTT. To overcome the aggregation and potential toxicity of PTAs, their integration into suitable carriers has been carried out, particularly for wound treatment (Maleki et al., 2021). Owing to their unique 3D porous structures, hydrogels effectively encapsulated PTAs to form a photothermal hydrogel. This not only enhances the uniform distribution of heat but also gives the damaged tissue a moist environment, which helps promote the healing process (Zhang et al., 2021).

Photothermal hydrogels offer unique advantages in the field of biomedicine. First, through photothermal effects, these hydrogels can precisely regulate the local temperature (Ma and Yan, 2021). This capability is crucial for meeting the requirements of different tissue regeneration stages, promoting cell proliferation and angiogenesis, and minimizing adverse effects on the surrounding healthy tissue (Jia et al., 2020). Second, photothermal hydrogels can combat infections by generating heat locally (Guo et al., 2023). This is key to preventing infections during tissue regeneration and maintaining a clean environment for wound healing. Finally, conveniently, photothermal hydrogels including traditional hydrogels can integrate drugs, growth factors, and other bioactive substances (Liu et al., 2019; Alinezhad et al., 2023). However, unlike traditional hydrogels, photothermal hydrogels achieve precise drug delivery via temperature control (Zhang et al., 2022). This precise drug delivery system contributes to the more accurate regulation of biological effects and plays a key role in the treatment process. Therefore, photothermal hydrogels ingeniously integrate the advantages of traditional hydrogels and photothermal effects. By designing different types of photothermal hydrogels, the precise treatment of tissues can be achieved.

In this review, we focus on the research into the development of photothermal hydrogels for controlling infections and promoting tissue regeneration. We first discuss the main types of PTAs, as well as the design concept and preparation method for photothermal hydrogels. The potential mechanisms underlying the photothermal effect on antibacterial activity, angiogenesis, and tissue reconstruction are summarized. In addition, photothermal hydrogels have excellent applications in controlling infection and repairing various tissue defects. Finally, we discuss the challenges and prospects of photothermal hydrogels in the treatment of infections and tissue regeneration. Overall, this review will be helpful for further preparation of more efficient photothermal hydrogels for infection control and tissue regeneration.

## 2 Main types of photothermal agents

PTAs are the core elements of PTTs, and, thus, the selection of the appropriate PTA is key to improving therapeutic effects. With the increase in demand for antibacterial agents and the continuous development of photothermal technology, PTAs have attracted considerable attention. In these systems, the heat generated by the photothermal effect not only inhibits bacterial growth but also promotes tissue regeneration, providing a dual treatment. Various materials have been studied as PTAs, and each has unique characteristics and applications. In the medical field, the most commonly used PTAs are either organic (OPTAs) or inorganic (IPTAs), as discussed in Sections 2.1, 2.2.

### 2.1 Organic photothermal agent

**FIGURE 1:**
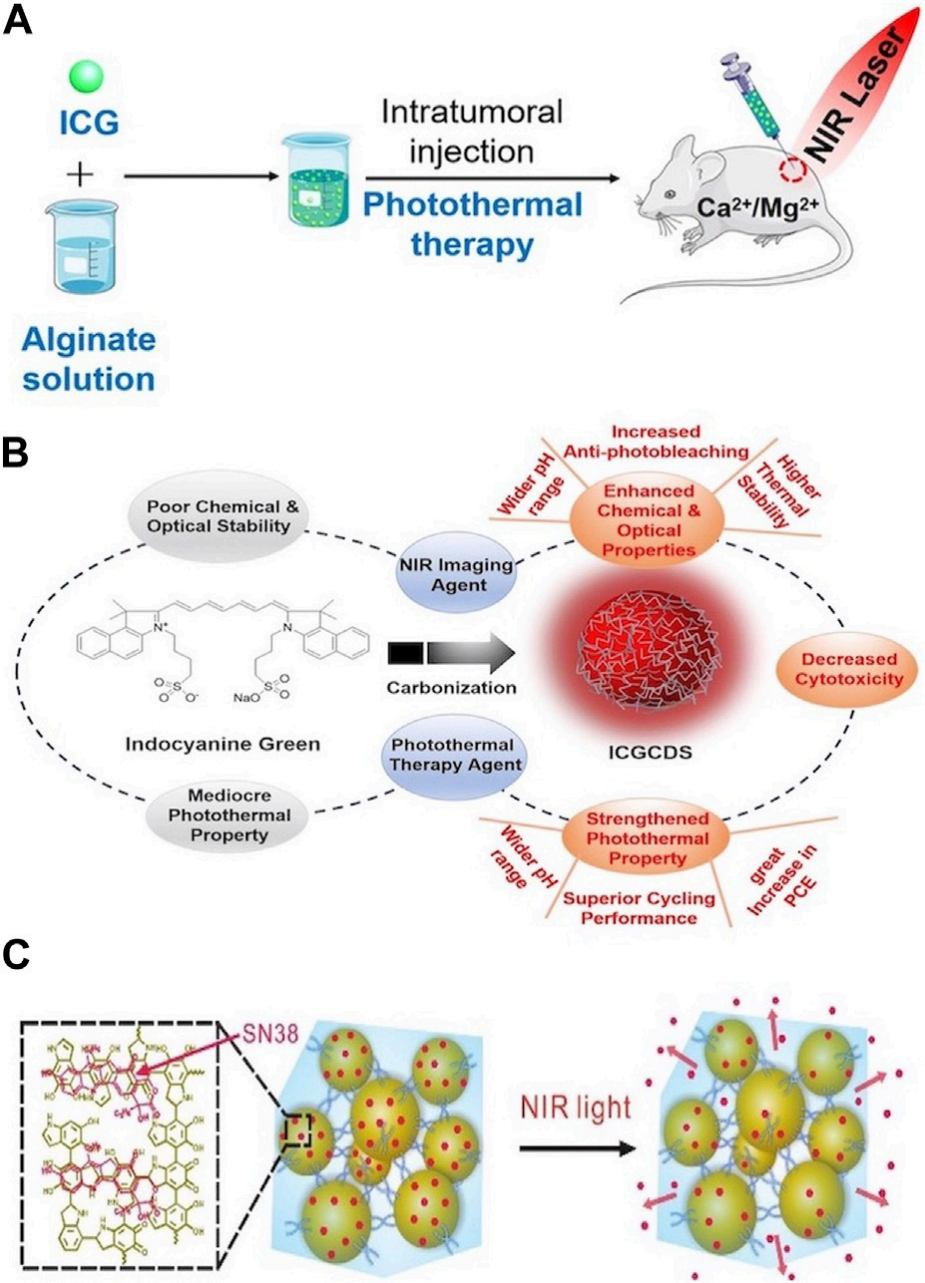
**(A)** Stimuli-Responsive Photothermal ICG−Alginate Hydrogel. Reproduced from ref (Pan et al., 2019) with permission from ACS Appl. Mater. Interfaces, copyright 2019. **(B)** Experimental design idea to prepare ICGCDs via the simple carbonization of ICG, which not only inherit the characteristics of ICG, but also refrain from its shortcomings. Reproduced from ref (Jiang et al., 2023) with permission from The Royal Society of Chemistry, copyright 2023. **(C)** Schematic represents SN38 loaded on PDANPs via π−π stacking and released from PDA/PEG hydrogel upon NIR irradiation. Reproduced from ref (Wang et al., 2017) with permission from Chemistry of Materials, copyright 2017.

OPTAs primarily include organic dyes (such as indocyanine green [ICG] and prussian blue [PB]) and polymer NPs (such as polydopamine [PDA] and polypyrrole [Ppy]). Most OPTAs exhibit good biocompatibility and degradability. For example, ICG, which fluoresces in the near-infrared spectral range, is a common organic dye nanomaterial (Egloff-Juras et al., 2019). Because of its low toxicity, it is widely used in medical imaging. In addition, Pan et al. prepared an *in situ* formed ICG-sodium alginate hydrogel. The hydrogel not only has excellent biocompatibility but also excellent ICG fixation ability. On light irradiation, ICG as a PTA can accumulate in the hydrogel, thereby reducing the adverse effects caused by the diffusion of ICG into surrounding tissue significantly (Figure 1A) (Pan et al., 2019). However, the chemical and optical stabilities of ICG are poor. To overcome these shortcomings, Jiang et al. developed a simple carbonization strategy for ICG and prepared nanosized ICG carbon dots (ICGCDs) using a simple one-step hydrothermal method. ICGCDs not only inherit the unique near infrared (NIR) emission and photothermal conversion ability of ICG but also significantly improve their chemical and photostability, photobleaching resistance, and biocompatibility. Compared to ICG, the photothermal conversion efficiency (PCE) of ICGCDs is nearly 50% higher (Figure 1B) (Jiang et al., 2023).

Unlike organic dyes, polymer NPs have good chemical and structural stabilities. PDA is a polymeric NIR-absorbing material that has attracted considerable attention recently. When PDA is introduced into hydrogels, their photothermal and antibacterial properties are enhanced. For example, a photothermal polydopamine–polyacrylamide/Mg^2+^ (PDA-PAM/Mg^2+^) composite hydrogel was synthesized. The incorporation of Mg^2+^ increased the thermal stability of the hydrogel, whereas PDA significantly improved the antibacterial effect through the a photothermal effect (Guo et al., 2022). Wang et al. designed and synthesized a PDA-NP-knotted polyethylene glycol (PEG) hydrogel loaded with 7-ethyl-10-hydroxycamptothecin (SN38). Considering the excellent PCE of PDA, the drug can be accurately released on demand under illumination (Figure 1C) (Wang et al., 2017).

### 2.2 Inorganic photothermal agent

**FIGURE 2:**
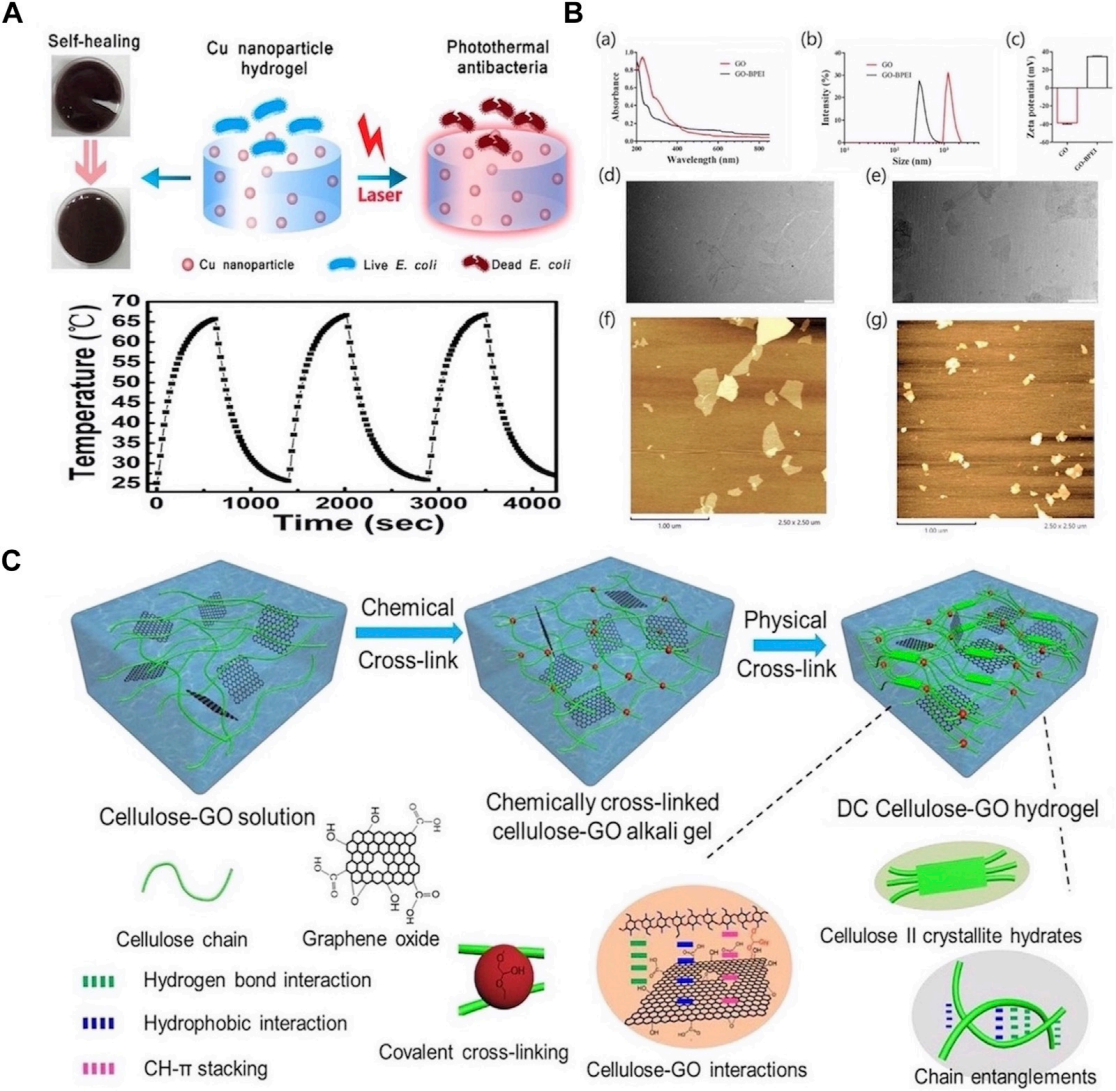
**(A)** The self-healing and photothermal properties of Cu-Nanoparticle Hydrogel and the temperature change of Cu-nanoparticle hydrogel over three cycles of laser irradiation on/off. The laser was operated at 660 nm and 1.5 W/cm^2^. Reproduced from ref (Chen et al., 2017) with permission from ACS Appl. Mater. Interfaces, copyright 2017. **(B)** Characterization of GO and GO-BPEI. (a) UV vis absorption spectra of GO and GO-BPEI aqueous dispersions. (b) Size distribution curves of GO and GO-BPEI. (c) Zeta potentials of GO and GOBPEI aqueous dispersions. TEM images of (d) GO and (e) GO-BPEI (Scale Bar = 1 μm). AFM images of (f) GO and (g) GO-BPEI. Reproduced from ref (Zhang X. et al., 2022) with permission from Elsevier, copyright 2022. **(C)** Schematic displaying the prepa ration of double-crosslinked cellulose/GO (DCCG) composite hydrogels. Reproduced from ref (Wei et al., 2022) with permission from Elsevier, copyright 2022.

IPTAs include metals (e.g., Ag, Cu, and Au) and carbon NPs (e.g., graphene oxide [GO] and carbon nanotubes). Metal NPs are widely used in PTT owing to their low production cost, excellent photothermal absorption performance, and broad-spectrum antibacterial properties. Recently, composite materials comprising metal NPs in hydrogels have attracted considerable attention. These composite materials exhibit good stability and maintain excellent photothermal performance under a range of illumination conditions. Chen et al. prepared Cu NPs using the polyol method and incorporated them into polysaccharide hydrogels to synthesize stable metal nanogels (NGs). The high photothermal conversion of the copper NPs enabled rapid heating under light irradiation, resulting in the hydrogel exhibiting good antibacterial properties. Moreover, the temperature of the composite hydrogel reached more than 65 °C after three cycles of laser irradiation, indicating that the hydrogel embedded with Cu NPs could withstand long-term repeated laser irradiation (Figure 2A) (Chen et al., 2017). In addition, the absorption of bacteria by metal NPs is important for their photothermal ablation activity. Studies have demonstrated that the modification of the surface of metal NPs with different functional groups can enhance their ability to absorb and destroy bacteria. Al-Bakri et al. prepared phospholipid-modified AuNP hydrogels, which significantly improved photothermal-induced cell destruction and lysis under laser irradiation (Al-Bakri and Mahmoud, 2019).

However, at higher concentrations, IPTAs easily aggregate, causing an uneven temperature distribution because of photothermal effects, which may impact tissue repair. GO is a typical type of carbon NP, and its surface can be easily modified. Thus, Zhang et al. developed an injectable self-healing hydrogel based on the photothermal effects. Notable, GO was used as a photothermal agent; after grafting with branched polyethyleneimine (BPEI), the GO was dispersed into small particles, which significantly improved its stability. In addition, Schiff bonds were formed between GO-BPEI and the hydrogel skeleton, which significantly enhanced the mechanical properties of the photothermal hydrogel (Figure 2B) (Zhang et al., 2022). Similarly, Wei et al. prepared cellulose-GO composite hydrogels via chemical and physical crosslinking. Covalent and noncovalent interactions between cellulose and GO significantly enhanced the strength and toughness of the hydrogel (Figure 2C). In addition, because of its excellent photothermal conversion ability, GO effectively converts infrared light into heat, thus inactivating and killing microorganisms (Wei et al., 2022).

In summary, although OPTAs have good biocompatibility with PTT, their photostability must be addressed. However, although IPTAs have excellent photothermal properties, they have problems such as easy agglomeration and potential biological toxicity. Practically, OPTAs and IPTAs are frequently combined into PTAs having complex structures to obtain the desired results. In this way, they achieve high stability and photothermal conversion efficiency while also having good biocompatibility and degradability.

## 3 Design principle and preparation of photothermal hydrogels

Photothermal hydrogels are formed by the complexation of PTAs and the hydrogel matrix. Because the interaction between PTAs and hydrogels forms the structural basis of photothermal hydrogels, the method of introduction of the PTAs into the hydrogel matrix is particularly important. Three major approaches for designing photothermal hydrogels based on different construction methods have been reported: 1) hybrid photothermal agents in hydrogels, 2) photothermal agents modified in the backbone of hydrogels, and 3) *in situ* formation of photothermal nanoparticles in hydrogels. Each approach is discussed in detail in the following sections.

### 3.1 Hybrid PTAs in hydrogels

**FIGURE 3:**
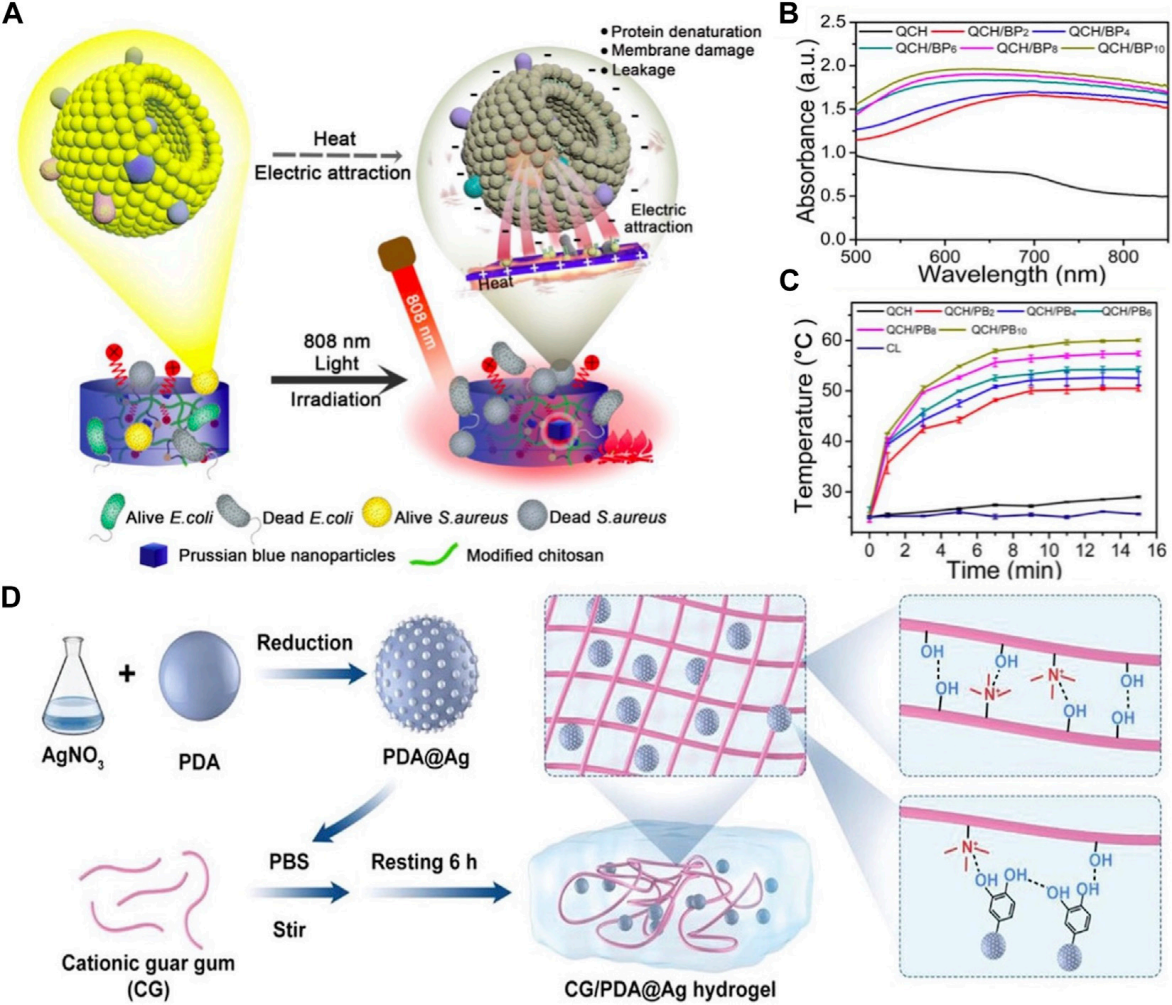
**(A)** Schema illustrating the antibacterial mechanism of the hydrogels. **(B)** The UV–vis spectrum; **(C)** The heating curves. Reproduced from ref (Han et al., 2020) with permission from Elsevier, copyright 2020. **(D)** Schematic illustrating the fabrication of CG/PDA@Ag hydrogel. Reproduced from ref (Qi et al., 2022) with permission from Wiley, copyright 2022.

The simplest and fastest method of producing photothermal hydrogels involves mixing monomers, polymers, and crosslinkers in suspensions of prefabricated PTAs. An advantage of this approach is that most inorganic and organic PTAs can be incorporated into photothermal hydrogels (Deng et al., 2021). For example, palladium nanosheets (Pd NSs) exhibit exceptional photothermal stability and strong optical absorption in the NIR region at 808 nm (Huo et al., 2021). Notably, they have been uniformly dispersed in a gelatin gel solution, and no aggregation was observed. Another advantage of this synthetic route is that the photothermal performance of the composite hydrogel can be controlled by regulating the PTA content. Han et al. reported that with the increase in the PB NP concentration, both the light absorption capacity and heating rate of the hybrid hydrogel increased (Figures 3A–C) (Han et al., 2020).

However, in monomer solution polymerization with crosslinkers and initiators, a tedious step involving thorough cleaning is required to remove excess monomers (Yang et al., 2018). Additionally, the high viscosity of hydrogel precursors prevents the effective mixing of PTAs. For example, polysaccharides, such as hyaluronic acid, sodium alginate, and chitosan, exhibit high viscosity owing to chain entanglement (Tang et al., 2019; Zhou et al., 2020; Zakerikhoob et al., 2021). Furthermore, several PTAs are hydrophobic and tend to agglomerate when mixed with polymers (Cao et al., 2022). In addition, the rapid deposition or aggregation of PTAs can affect the effectiveness of PTT. Therefore, the surface modification of PTAs is necessary before their incorporation in hydrogels. Li et al. used PDA and cationic guar gum (CG) to create a photothermally responsive composite hydrogel. However, PDA tends to agglomerate because of its surface-active groups. To prevent the aggregation of PDA, Ag NPs were grown on the PDA surface using the reduction–precipitation method (Figure 3D). This not only enhanced the stability of the hydrogel but also improved its photothermal conversion ability (Qi et al., 2022).

### 3.2 PTA modification of the hydrogel backbone

**FIGURE 4:**
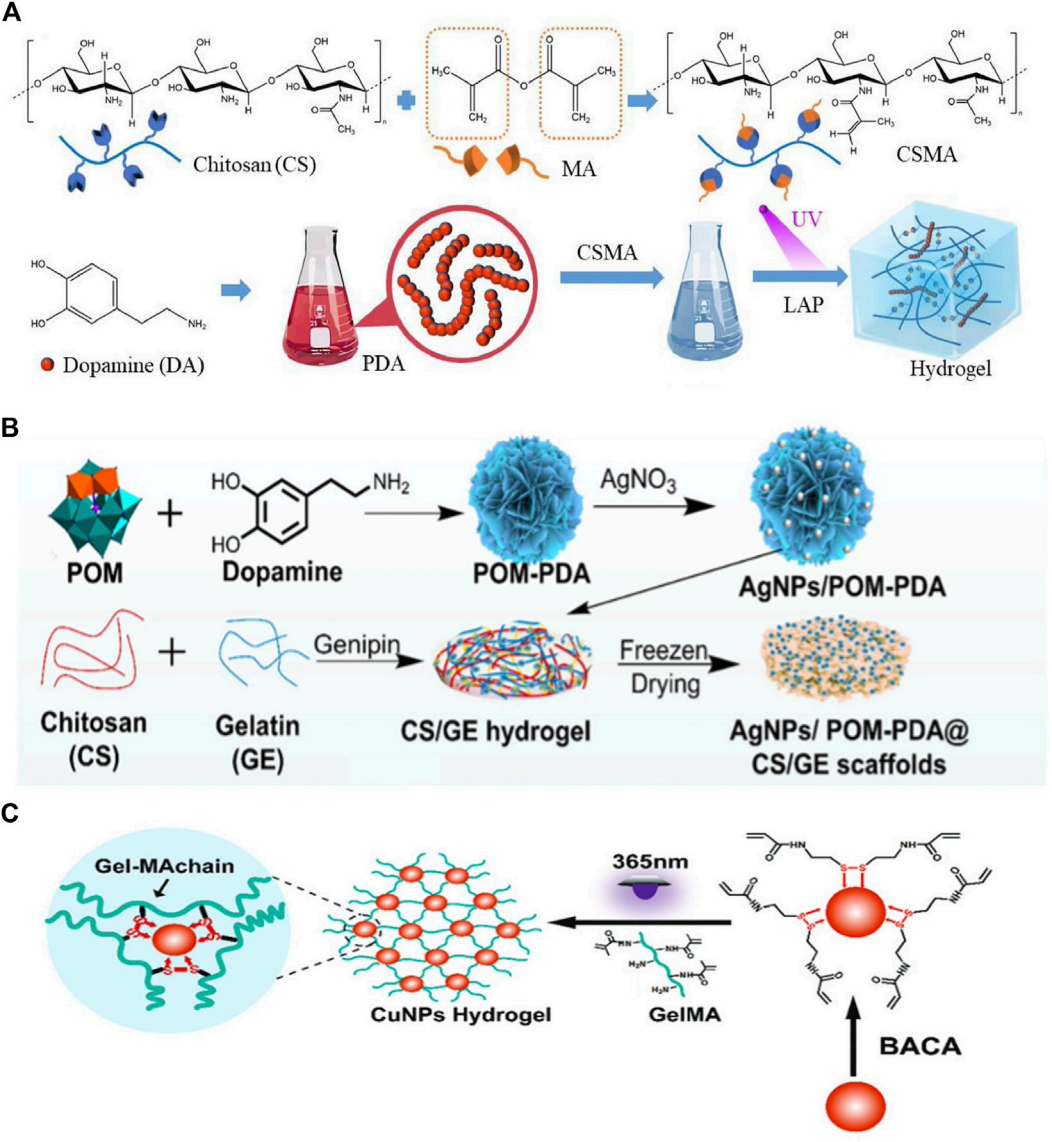
**(A)** Diagram of the method used to prepare CSMA/PDA injectable hydrogels. Reproduced from ref (Cheng, 2023) with permission from Elsevier, copyright 2023. **(B)** Schematic representation of hybrid AgNPs/POM-PDA nano-flowers-loaded CS/GE hydrogel scaffolds. Reproduced from ref (Zhou et al., 2022) with permission from Elsevier, copyright 2022. **(C)** Schematic formation of Cu-NP-embedded hydrogels. Reproduced from ref (Tao et al., 2019) with permission from The Royal Society of Chemistry, copyright 2019.

In this approach, monomers or polymers are premixed with crosslinkers to form a hydrogel matrix, followed by the modification of the hydrogel backbone with PTAs through covalent or other strong bonds. For instance, Cheng et al. first synthesized methacrylated chitosan (CMCS) as a hydrogel matrix and then incorporated PDA into the CMCS hydrogel via free radical polymerization (Figure 4A) (Cheng, 2023). Similarly, Zhou et al. developed a multifunctional hybrid hydrogel by integrating Ag nanoparticles/phosphotungstic acid-polydopamine nanoflowers (AgNPs/POM-PDA) into chitosan (CS)/gelatin (GE) hydrogels via the Schiff base reaction and Michael addition (Figure 4B). The incorporation of PTAs into this hydrogel conferred excellent photothermal and antibacterial properties (Zhou et al., 2022). However, there are several synthetic challenges, particularly with IPTAs, such as metal NPs, which may aggregate within the hydrogel because of their high surface charge (Shrestha et al., 2020).

As a result of the introduction of PTAs after hydrogel formation, the cross-linking density between the PTAs and hydrogel matrix can be low. Consequently, several IPTAs may be lost from the hydrogel matrix. If a large amount of an inorganic PTA is released to surrounding tissue, toxic effects can occur, and the photothermal properties of the hydrogel are reduced (Tang et al., 2023). To improve the stability of PTAs, Tao et al. chelated *N*,*N*-bis(acryloyl)cystamine (BACA) onto Cu NPs before preparing a Cu-NP-embedded photothermal hydrogel (Figure 4C). The formation of the Cu-S bond resulted in a more robust connection between the Cu NPs and the methacrylate gelatin (GelMA) hydrogel, leading to excellent photothermal properties under 808-nm laser irradiation (Tao et al., 2019).

### 3.3 *In situ* formation of photothermal NPs in hydrogels

Under certain conditions, PTAs can be formed *in situ* within hydrogels. In this method, PTAs are obtained through a series of reactions between their precursors and monomers or polymers, enabling their deep integration into the 3D network of the hydrogel. The primary advantage of *in situ* formation is the significant enhancement of the mechanical properties of the photothermal hydrogel. Furthermore, the uniform distribution of PTAs in the hydrogel matrix effectively improves the photothermal properties of the composite hydrogels.

**FIGURE 5:**
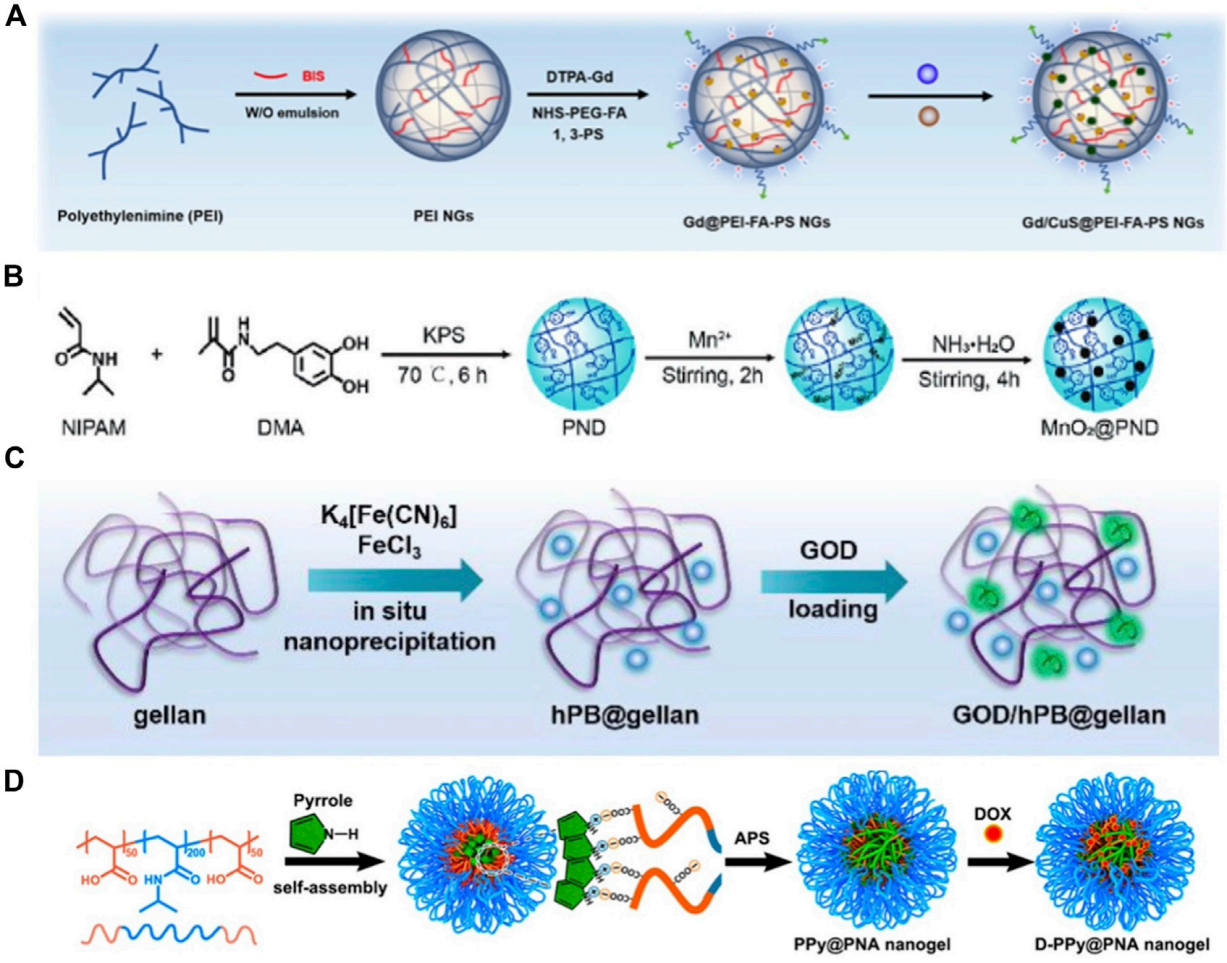
**(A)** Schematic Illustration of the Preparation of Gd/CuS@PEI-FA-PS NGs. Reproduced from ref (Zhang et al., 2020) with permission from ACS Appl. Mater. Interfaces, copyright 2020. **(B)** Schematic showing the preparation of the PND and MnO_2_@PND nanogels. Reproduced from ref (Fan M. et al., 2021) with permission from Wiley, copyright 2021. **(C)** Illustration of the process for the formation of GOD/hPB@gellan hydrogel. Reproduced from ref (Hao et al., 2020) with permission from The Royal Society of Chemistry, copyright 2020. **(D)** Schematic Illustration of the Preparation of D-PPy@PNA Nanogels. Reproduced from ref (Geng et al., 2020) with permission from ACS Appl. Mater. Interfaces, copyright 2020.

A common type of inorganic PTA, metal NPs can be generated in photothermal hydrogels through *in situ* polymerization of their precursors. In recent years, the *in situ* synthesis of metal NPs using NGs as nanoreactors has gained prominence. Zhang et al. developed polyethylenimine (PEI) hydrogels loaded with CuS NPs (Figure 5A). The functionalized PEI NGs can react with Cu^2+^ and S^2-^ to generate smaller CuS NPs *in situ*, imparting a hybrid hydrogel with outstanding photothermal properties (Zhang et al., 2020). Similarly, Fan et al. synthesized poly (*N*-isopropylacrylamide-*co*-dopamine methacrylamide) (PND) NGs through sediment polymerization, utilizing active functional groups, such as phenolic hydroxyl and carbonyl groups, on the surface to interact with Mn^2+^ and construct PND-Mn^2+^ complexes. Ultimately, the *in situ* mineralization of MnO_2_ NPs in PND NGs was achieved in an alkaline environment (Figure 5B) (Fan et al., 2021). The NPs prepared using this method exhibited controllable size and high stability.

Several OPTAs (such as PB and Ppy) can also be generated *in situ* within NGs via nanoprecipitation or inorganic reduction. Hao et al. combined PB NPs and glucose oxidase (GOD) to prepare a composite nanozyme@hydrogel. Specifically, PB NPs were synthesized in gellan gum using an *in situ* nanoprecipitation method, and the formed NPs had an extremely small size of 10 nm (Figure 5C). Moreover, PB NPs, which are typical PTAs, demonstrated good photothermal PCE under NIR irradiation (Hao et al., 2020). Later, Geng et al. prepared temperature-sensitive poly (acrylic acid-*b*-N-isopropylamide-*b*-acrylic acid/polypyrrole) (Ppy@PNA) NGs via the redox polymerization of pyrrole monomers in PNA micelles using ammonium persulfate (APS) as an oxidizing agent (Figure 5D) (Geng et al., 2020). Notably, the residual monomers and initiators in the reaction may render the composite hydrogel toxic, necessitating thorough cleaning to remove excess monomers.

In summary, the preparation of photothermal hydrogels can be divided into three main types: 1) the introduction of hybrid PTAs into hydrogels, similar to physical mixing and relies on non-covalent forces to achieve binding (Chang et al., 2023). This approach is suitable for scenarios involving simple operations, although the bonding strength is relatively low. 2) The modification of the hydrogel backbone with PTAs via covalent or other strong bonds (Zhou et al., 2023). This ensures that the PTAs are integrated into the hydrogel backbone, making them suitable for applications requiring high stability. 3) The *in situ* formation of photothermal NPs in hydrogels, in which PTAs are formed directly during hydrogel preparation, which may involve the formation of chemical bonds (Thies et al., 2018). For example, in the sol-gel process, chemical reactions promote the strong binding of photothermal NPs to the hydrogel. This method is applicable to scenarios requiring a higher binding strength. Therefore, when choosing this method, it is necessary to consider the properties of PTAs and hydrogels, as well as the requirements for specific applications.

## 4 Photothermal mechanism for infection control and tissue regeneration

The photothermal effect involves the conversion of light energy into heat. In recent years, it has been widely used for infection control and tissue regeneration. To understand the possible uses of the photothermal effect in these areas and enhance its healing impact and process, it is essential to investigate the precise mechanism of the photothermal effect for controlling infections and promoting tissue repair.

### 4.1 Antibacterial

The control of bacterial infections has always been an important medical challenge. The advent of the antibiotic-era was a defining point in the control and treatment of bacterial diseases. However, the long-term and excessive use of antibiotics has resulted in the development of bacterial resistance, which has become an increasingly prominent problem. Therefore, the study of low-dose treatments without antibiotics or precise administration has drawn attention. As a new and alternative treatment modality, PTT has numerous advantages, such as strong specificity, few side effects, and simple operation. Thus, it has significant potential for the treatment of localized lesions caused by bacteria.

For numerous bacteria, high temperatures cause cell death. Therefore, on illumination at a specific wavelength, heating can damage or kill bacteria. Crucially, the heat damages the bacterial cellular structure, including the membrane lipid layers, proteins, and nucleic acids, resulting in the loss of biological activity and ultimately cell death. For example, Teng et al. exploited the absorption capacity of gold nanocrosses for NIR light and the resulting PTT, which produced a local temperature of 60 °C, reduced the survival rate of *Pseudomonas aeruginosa* to 15% after irradiation for 10 min. Following irradiation at 70 °C for 5 min, all bacteria were inactivated. Notably, the bacterial biofilm, which is often more resistant to treatment than isolated bacteria, was clearly observed to be completely destroyed under confocal imaging (Teng et al., 2016).

However, for photothermal antibacterial therapy to be effective, temperatures higher (60 °C) than physiological temperature (37 °C), which can damage healthy cells and tissue around the treatment site, are required. Therefore, the development of PTT at 50 °C has become a focus. For example, Zhao et al. demonstrated that treatment at mild-mid temperatures also has an antibacterial effect. Specifically, the cell membranes of *Escherichia coli* and *Staphylococcus aureus* appear wrinkled and porous after NIR irradiation, suggesting that the integrity of the cell membrane had been reduced (Zhao et al., 2023). Similarly, Lin et al. constructed a bacterial-based multifunctional hydrogel that achieved high-efficiency low-temperature photothermal sterilization. Furthermore, its *in vivo* antibacterial efficiency reached an impressive 98.2% (Lin et al., 2021).

### 4.2 Angiogenesis

PTAs can also enhance the proliferation and migration of vascular endothelial cells, thereby promoting the formation of new blood vessels, which is crucial for supplying sufficient nutrients and oxygen to tissue for cell proliferation, metabolism, and repair, thus promoting the regeneration and repair of damaged tissue. Bioactive glass (BGs) has attracted considerable attention in the field of tissue repair because of its excellent biological activity. Specifically, the Si ions released by its decomposition can increase gap junction communication between human umbilical vein endothelial cells (HUVECs) and promote the production of angiogenic genes, thereby promoting the blood vessel growth (Kong et al., 2018). Additionally, Fe^3+^, which is biologically active, contributes to angiogenesis and wound healing (Zhang et al., 2021). Notably, the combination of Fe^3+^ doped BGs and hydrogels can effectively increase the total vascular length and number of vascular branches under photothermal conditions, thus increasing angiogenesis (Hou et al., 2023).

**FIGURE 6:**
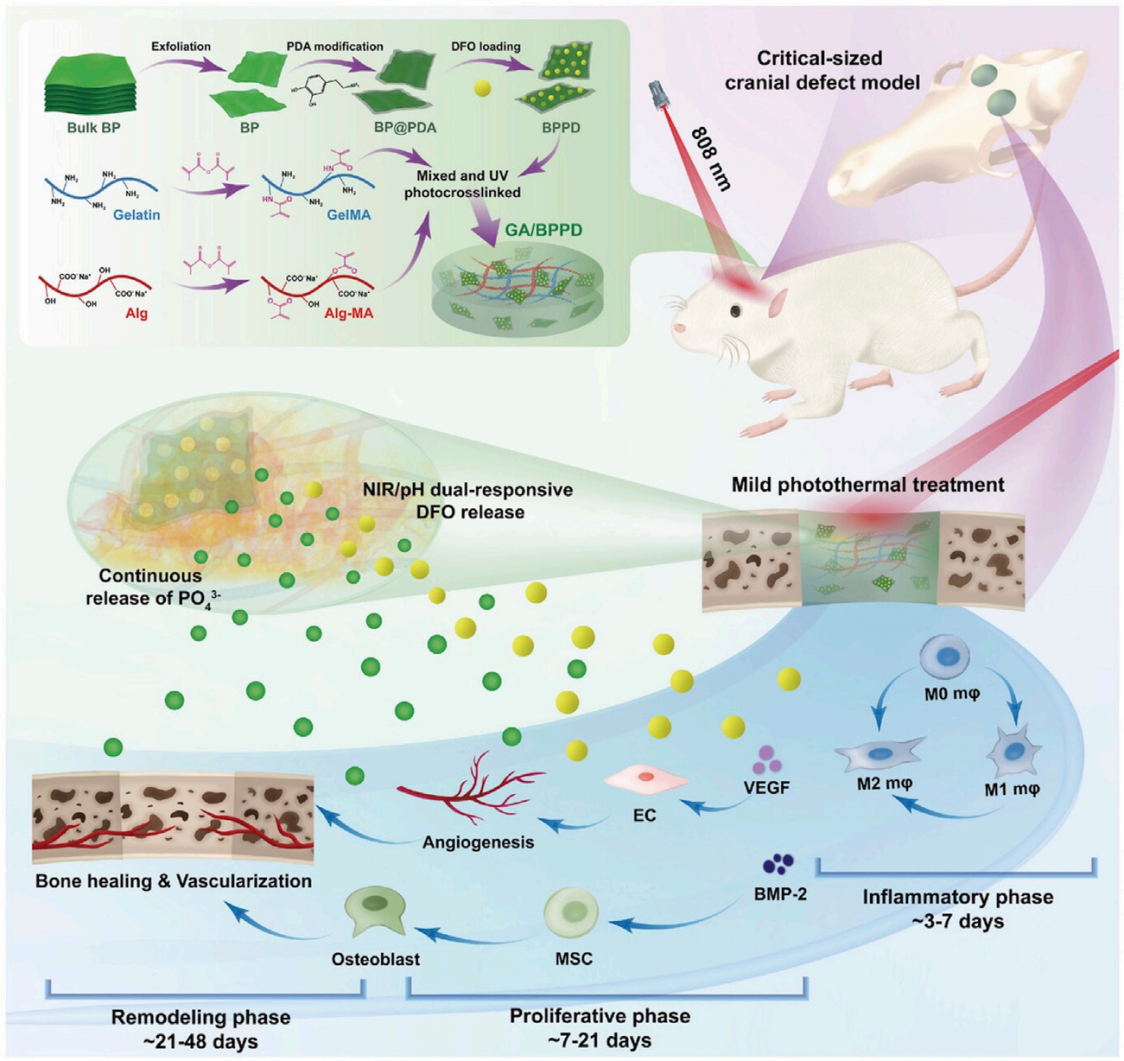
Schematic illustration for fabrication and application of smart-responsive multifunctional therapeutic system with mild photothermal activity for augmented bone regeneration through spatiotemporal manipulation of the immune microenvironment, stem cell recruitment and vascular development, and osteogenic differentiation throughout the whole healing process. Reproduced from ref (Wu et al., 2024) with permission from Wiley, copyright 2024.

Angiogenesis is also involved in immune response regulation, helping to remove pathogens and waste from damaged tissues while promoting inflammatory regulation during repair. Recently, Wu et al. successfully prepared GA BPPD hydrogels by encapsulating polydopamine-decorated deferoxamine (DFO)-loaded black phosphorus nanosheets (BPPD) in gelatin methacrylate/sodium alginate methacrylate (GA). Under mild NIR light irradiation conditions, the hydrogel induced macrophage polarization toward the M2 phenotype and promoted anti-inflammatory activity, angiogenesis, and release of osteoblast factors, thereby enhancing angiogenesis and attracting endogenous stem cells, which are key to the early stages of tissue healing (Figure 6) (Wu et al., 2024).

During angiogenesis, a variety of growth factors and cell signaling molecules, such as vascular endothelial growth factor (VEGF) and basic fibroblast growth factor (bFGF), are released. These factors can directly affect the proliferation, migration, and differentiation of the surrounding cells, thereby promoting tissue regeneration. Xu et al. prepared PDA/copper-doped calcium silicate ceramic (Cu-CS) composite hydrogels. On irradiation, Cu-CS upregulated the expression level of hypoxia-inducible factor-1α (HIF-1α) in human dermal fibroblasts (HDFs). The overexpression of HIF-1α further increased the expression of VEGF in HDFs. Subsequently, high expression of VEGF affects co-cultured human umbilical vein endothelial cells (co-HUVECs) and initiates angiogenesis (Xu et al., 2020).

### 4.3 Tissue reconstruction

Crucially, the local temperature produced by the photothermal effect can be modulated by adjusting light irradiation parameters, and the selection of an appropriate temperature can promote cell activity and metabolism. In the moderate-temperature (40°C–42 °C) range, the metabolic activity of cells increases, including protein synthesis and cell signal transduction, thereby promoting tissue regeneration. Xie et al. developed a treatment platform that allowed the visual observation of wounds and their complications. In the early healing stage, they found that the photothermal hydrogels significantly promoted the formation of new blood vessels. However, observations at a later stage demonstrated a significant decrease in the number of blood vessels, indicating that the vascular tissue had been replaced by fibroblasts and extracellular matrix, which effectively promoted wound healing (Xie et al., 2022).

α-Lipoic acid (LA) has excellent anti-inflammatory effects; for example, it can reduce the levels of various inflammatory factors, such as tumor necrosis factor-αlpha (TNF-α), interleukin-6 (IL-6), and interleukin-1β (IL-1β), by inhibiting the expression of nuclear factor-kappa-B (NF-κB) (Alleva et al., 2005; Shay et al., 2009; Nasole et al., 2014). Luo et al. developed a NIR light-excited hydrogel containing LA-modified Pd nanoparticles (PdNPs). The hydrogel exhibited excellent biocompatibility, did not damage skin repair-related cells, and could be used as a flexible tissue repair platform to promote cell activity, such as cell adhesion and proliferation. High cellular activity accelerates skin tissue regeneration and wound healing. In addition, the hydrogel reduced the levels of reactive oxygen species (ROS) and inflammatory factors at the wound site, thereby shortening the inflammatory period. Moderate shortening of the inflammatory phase helps advance the proliferative phase, thus promoting effective repair of skin defects (Luo et al., 2022).

In summary, the photothermal effect as an innovative treatment and repair method is of significant importance for infection control and tissue regeneration. Thus, the development of an in-depth understanding of the mechanism underlying the photothermal effect on infections and tissues will provide an important theoretical basis and technical support for research and clinical applications in related fields.

## 5 Application of photothermal hydrogels in infection control and tissue regeneration

Hydrogels can be used for tissue regeneration because of their good biocompatibility, hydrophilicity, biodegradability, and similar 3D porous structure to that of the ECM. Moreover, hydrogels with photothermal ability, as multifunctional biomaterials, show increased bactericidal effects owing to the incorporation of PTAs compared to neat hydrogels. However, the photothermal effect can not only control infection but also promote tissue regeneration. Because of these excellent properties, photothermal hydrogels have been applied for the treatment of many types of infected tissue (Table 1). In this section, we introduce the applications of photothermal hydrogels in infection control and tissue regeneration in detail.

**TABLE 1:** Application of photothermal hydrogels in the treatment of infected tissues.

Hydrogels	Photothermal agents	Light and time	Bacteria	Application	References
GelMA-Au NBPs@SiO_2_	Au NBPs	808 nm 1.2 W/cm^2^ 5 min	P. gingivalis	Antibacterial Drug release	Lin et al. (2020a)
CS/HC/HA/BP	BP	808 nm 1.5 W/cm^2^ 10 min	*E. coli* + *S. aureus*	Antibacterial Bone regeneration	Zhao et al. (2023b)
GA-Ag NP/Carrageenan	GA-Ag NPs	808 nm 1.5 W/cm^2^ 10 min	*E. coli* + *S. aureus*	Antibacterial Wound healing	Liu et al. (2020b)
BP@CAu	AuNRs	808 nm 1.0 W/cm^2^ 6 min	*S. aureus* + *P. aeruginosa*	Antibacterial Wound healing	Jia et al. (2023)
QCS/OD/TOB/PPY@PDA	PPY@PDA	808 nm 1.4 W/cm^2^ 10 min	MRSA	Antibacterial Wound healing	Huang et al. (2022b)
Ti-RP/PCP/RSNO	PDA	808 nm 1.0 W/cm^2^ 10 min	MRSA	Antibacterial Bone regeneration	Li et al. (2020)
SrCuSi_4_O_10_/GelMA	SrCuSi_4_O_10_	808 nm 1.0 W/cm^2^ 5 min	S. mutans + L. casei	Antibacterial Pulp regeneration	Qiu et al. (2023)
HA/PEGSB/CMP	CMP	808 nm 0.8 W/cm^2^ 3 min	*E. coli* + MRSA	Antibacterial Wound healing	Li et al. (2022a)
QCS/MA/PVP/DA	PDA	808 nm 0.8 W/cm^2^ 5 min	*E. coli* + *S. aureus*	Antibacterial Wound healing	Xu et al. (2023)
Met@CuPDA NPs/HG	CuPDA NPs	808 nm 0.5 W/cm^2^ 10 min	MRSA + *E. coli* + *S. aureus*	Antibacterial Wound healing	Zhu et al. (2023)
P(NIPAM-AM)/MeO-TSI@F127 NPs	MeO-TSI@F127 NPs	808 nm 1.5 W/cm^2^ 10 min	MRSA	Antibacterial Wound healing	Fu et al. (2023)
NOCS/OSA/FA	FA (Fe_2_SiO_4_)	808 nm 0.36 W/cm^2^ 10 min		Angiogenesis Wound healing	Sheng et al. (2021)
GelMA/PMMA/PDA	PDA	808 nm 0.99 W/cm^2^ 5 min		Bone regeneration	Wu et al. (2022)
AMAD/MP	MXene@PDA NSs	808 nm 1.0 W/cm^2^ 5min	*E. coli* + *S. aureus*	Osteogenic Antibacterial	Wu et al. (2023)
CS/rGO	rGO	808 nm 0.5 W/cm^2^ 10 min		Bone regeneration	Wang et al. (2021b)
DHCP-10PIP/d	ICG	808 nm 2.0 W/cm^2^ 10 min		Bone regeneration	Kuang et al. (2021)

### 5.1 Infection control

Common types of clinical trauma are skin and bone injuries. Under normal circumstances, wound recovery comprises four stages: hemostasis, inflammation, proliferation, and remodeling (Yu et al., 2019). However, external environmental factors, particularly infection by pathogens, can impede wound healing, resulting in chronic wounds that have a detrimental effect on quality of life (Kang et al., 2023). In recent years, various dressings have been developed to control infections, promote hemostasis, and enhance angiogenesis (Dong and Guo, 2021). As wound dressings, hydrogels are popular because of their moisturizing properties, biocompatibility, and biodegradability (Wang et al., 2022b). In addition, hydrogels with photothermal properties can effectively eradicate bacteria through local heating. (Zhang et al., 2023; Zheng and Xiao, 2023).

As discussed, antibiotics are widely used to treat bacterial infections (Fan et al., 2021), which has resulted in the development of drug-resistant bacteria and complicated wound treatment (Xu et al., 2022). As a new type of non-invasive treatment, PTT demonstrates significant potential for treating drug-resistant bacteria and bacterial biofilms. For example, Lin et al. incorporated mesoporous silica-coated Au nanobipyramids (Au NBPs@SiO_2_) and minocycline into GelMA. The resulting composite hydrogels exhibited controlled drug release under 808-nm NIR irradiation. Simultaneously, the combination of antibiotics and photothermal therapy not only removed residual bacteria in the early stages of treatment but maintained the bacteria at a low level, demonstrating excellent antibacterial effects (Lin et al., 2020).

**FIGURE 7:**
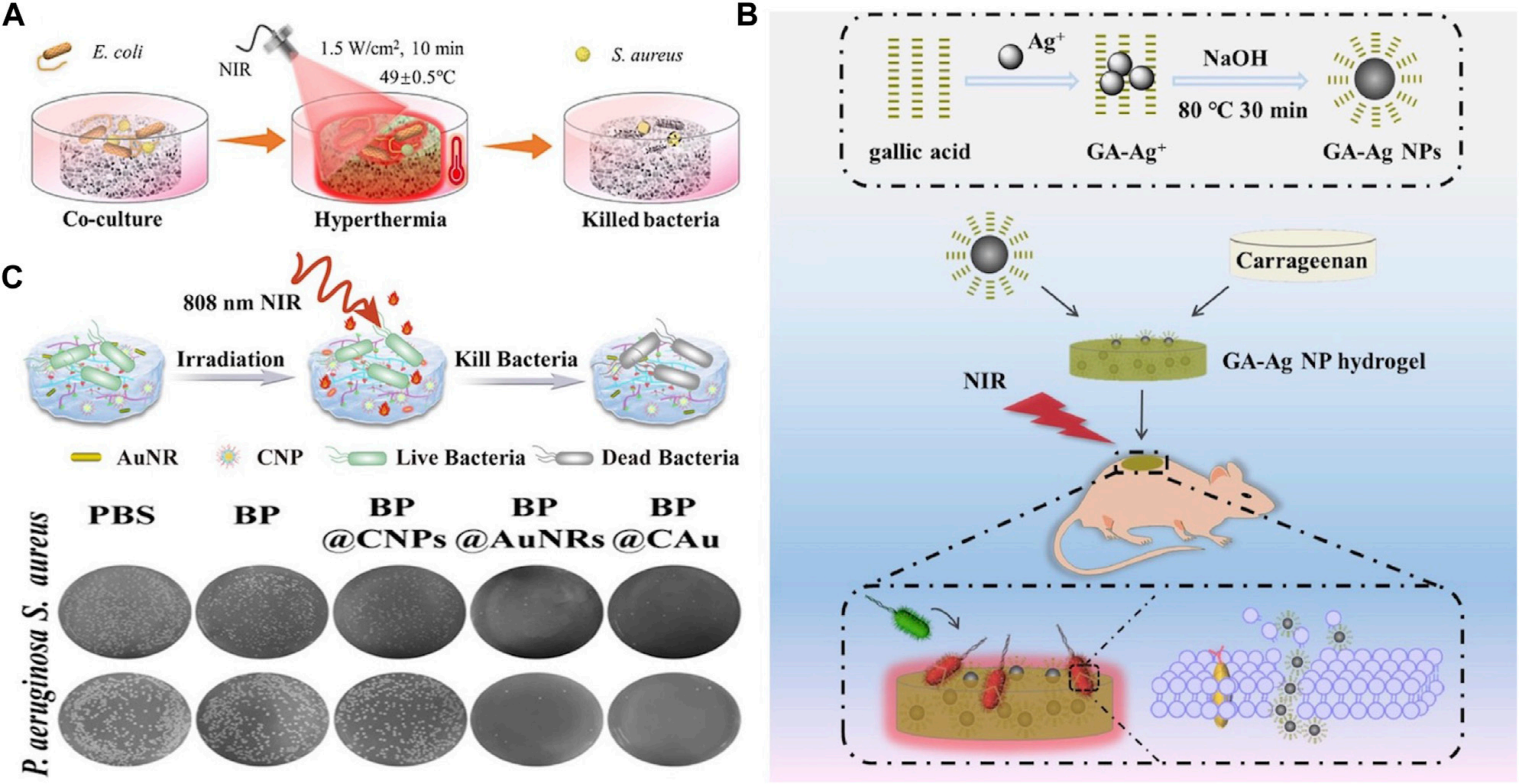
**(A)** Schematic illustration of the antibacterial process of the CS/HC/HA/BP scaffold with NIR irradiation. Reproduced from ref (Zhao Y. et al., 2023) with permission from Elsevier, copyright 2023. **(B)** Schematic illustrates of the synthesis of GA-Ag NPs and their incorporation into a hydrogel, forming a combined system capable of synergistically eliminating bacteria through photothermal and innate bactericidal effects. Reproduced from ref (Liu Y. et al., 2020) with permission from Elsevier, copyright 2020. **(C)** Antibacterial effect of BP@CAu hydrogels. Reproduced from ref (Jia et al., 2023) with permission from ACS Appl. Mater. Interfaces, copyright 2023.

A key feature of photothermal hydrogels is the ability to control the temperature precisely, thus enabling the treatment of wounds infected with bacteria (Yi et al., 2021). As discussed, on heating, bacterial proteins are denatured, and the bacterial cell membrane is destroyed, thereby inhibiting growth. Zhao et al. introduced a chitosan/hydroxypropyltrimethyl ammonium chloride chitosan/hydroxyapatite/black phosphorus (CS/HC/HA/BP) composite hydrogel with multi-stage PTT, and different therapeutic effects were achieved through the control of the temperature. Briefly, tumors and bacteria could be targeted at 49°C ± 0.5 °C (Figure 7A), whereas bone tissue regeneration was promoted at 42°C ± 0.5 °C (Zhao et al., 2023). In addition, Liu et al. integrated gallic acid-functionalized silver NPs (GA-Ag NPs) with carrageenan to create an antibacterial GA-Ag NP hydrogel (Figure 7B). Following 10 min of NIR irradiation, the hydrogel temperature increased from 17.2°C to 49.9°C, effectively killing bacteria and achieving the sustained release of Ag^+^, which also shows a bacterial effect (Liu et al., 2020). Similarly, Jia et al. constructed a microenvironment-adaptive hydrogel, BP @ CAu by integrating gold nanorods (AuNRs) and curcumin nanorods (CNPs), in which AuNRs with a high photothermal conversion ability were used as PTAs. The BP@CAu hydrogel can be rapidly heated to 60 °C under 808-nm NIR irradiation, and the wound site has nearly no bacteria on the agar plate (Figure 7C) (Jia et al., 2023).

Interestingly, the acidic compounds produced during bacterial growth can trigger the release of the antibiotic tobramycin (TOB) on demand, thereby avoiding the indiscriminate use of antibiotics. For example, Huang et al. developed a range of self-healing hydrogels with smart TOB release in response to bacterial growth. Following 10 min of *in vivo* PTT, significant bactericidal activity was observed against drug-resistant bacteria. (Huang et al., 2022). In addition, during wound treatment, certain concentrations of nitric oxide (NO) can exhibit excellent antibacterial properties and tumor-cell-killing effects (Schäffer et al., 1996; Hoang Thi et al., 2018; Liu et al., 2020; Liu et al., 2023). Li et al. demonstrated that RP-modified Ti implants/CS and PDA modificatory poly (vinyl alcohol) (PVA)/NO release donor (Ti-RP/PCP/RSNO) hydrogels can achieve controlled NO release under NIR irradiation. The combination of hyperthermia and released NO demonstrated excellent antibacterial properties. In a crystal violet (CV) assay, the Ti-RP/PCP/RSNO group exhibited a higher methicillin-resistant *Staphylococcus aureus* (MRSA) biofilm eradication ratio (over 93.1%) compared to the Ti-RP group (32%) and Ti-RP/PCP groups (45.6%) (Li et al., 2020).

**FIGURE 8:**
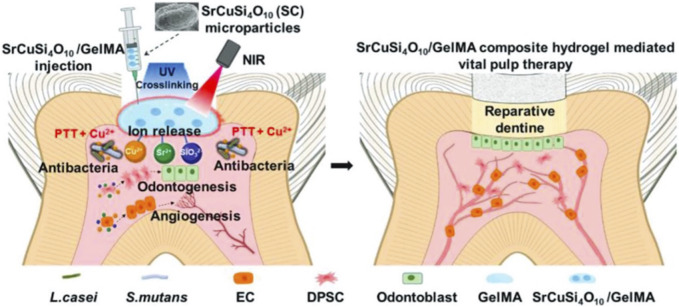
Schematic illustration of the application of SC/Gel composite hydrogel and NIR irradiation for infected dental pulp treatment, through releasing Sr^2+^, Cu^2+^, and SiO_3_^2−^ ion in response to the promoted antibacterial effect, angiogenesis, and odontogenesis. Reproduced from ref (Qiu et al., 2023) with permission from Wiley, copyright 2023.

Infectious tissue defects include not only those of flesh and bone but also extend to periodontal and corneal tissue, which are susceptible to bacterial infection. PTT has been used to treat infectious pulpitis and bacterial keratitis. Root canal work is the primary clinical treatment for pulpitis (Lu et al., 2020). However, after removing the infected pulp, the tooth tissue lacks nutrients and can become brittle or fracture, even though the pulp has a good regenerative ability (Moussa and Aparicio, 2019). Vital pulp therapy (VPT) is a conservative treatment that avoids the adverse effects of root canal procedures (Iaculli et al., 2022). To enhance the therapeutic effect of VPT, Qiu et al. used a GelMA hydrogel loaded with SrCuSi_4_O_10_ (SC) to create an SC/Gel composite hydrogel. Having the sustained release of Sr^2+^, Cu^2+^, and SiO_3_^2−^, this composite hydrogel demonstrates excellent NIR photothermal conversion ability, effectively eliminating bacteria and inhibiting biofilm formation. Rat dental pulp stem cells (rDPSCs) and HUVECs were used to study the effect of SC on odontogenesis and angiogenesis and to explore its possible molecular mechanisms (Figure 8) (Qiu et al., 2023).

In addition to its role in vision, the cornea has a protective role (Xiong et al., 2019). However, bacterial keratitis not only damages the corneal tissue but causes eye infections and even blindness. Currently, corneal transplants are used to treat such corneal diseases; however, this process has several disadvantages, including high costs, donor shortages, and risk of graft rejection (Armitage et al., 2019; Singh et al., 2019; Ang et al., 2020; Yin, 2021). Considering their outstanding properties, particularly their ability to mimic the corneal stromal cell matrix, hydrogels are promising multifunctional materials for corneal repair (Kong et al., 2020; Khosravimelal et al., 2021; Shen et al., 2023). Meng et al. designed a versatile composite hydrogel (SQPV) fabricated from glycidyl methacrylate-functionalized quaternized chitosan (QCSG) and methacrylate silk fibroin (SFMA). The hydrogel formed a double-layer network structure under light irradiation, providing good anti-inflammatory, antibacterial, proliferative, and tissue-remodeling functions. Moreover, the antibacterial activity of QCSG in SQPV helped to eliminate bacterial contamination around corneal tissue (Meng et al., 2023).

### 5.2 Tissue regeneration

Photothermal treatment of damaged tissue is not limited to infection control but can also promote tissue regeneration and repair. Of note, hydrogels have excellent adhesion and shape conformity, and can be attached to the surface of tissues or wounds, thus providing a platform to support cell adhesion and growth. The photothermal hydrogel absorbs light energy and converts it into heat, resulting in a local temperature increase. Moderate thermal effects can stimulate cell activity including cell proliferation, differentiation, and collagen production, thereby promoting tissue regeneration. Photothermal hydrogels can also control the temperature to ensure that the temperature stimulation effect on the tissue is within an appropriate range. In addition, photothermal hydrogels can assist in tissue regeneration by improving blood circulation, regulating inflammatory responses, and promoting cell activity.

Skin wounds are typically categorized as acute or chronic (Raziyeva et al., 2021). Typically, when the body experiences acute trauma, the wound healing mechanism is spontaneously initiated and progresses until the skin tissue structure and function are fully restored (Hao et al., 2022). However, the frequent movement and stretching of the wound site cannot be avoided during the healing process of damaged tissue, and even acute wounds that would normally heal rapidly may suffer prolonged healing times (Li et al., 2022). Composite hydrogel dressings have been proposed to address this challenge. For instance, hyaluronic acid/poly (ethylene glycol)-*co*-poly (glycerol sebacate)/cuttlefish melanin nanoparticle (HA-PEGSB-CMP) hydrogels have demonstrated excellent tissue adhesion. The reaction between PEGSB and HA, along with the cross-linking of CMP with HA, imparts a double-dynamic Schiff base network to the hydrogel. Notably, when the hydrogel sheet was cut into two semicircles, the self-healing efficiency reached up to 102% and the stretchability exceeded 200%. In addition, these hydrogels could sterilize the wound site and promote healing via PTT (Li et al., 2022).

Frequent movement can also create irregularities in wounds, increasing the complexity of healing and treatment. Therefore, hydrogel dressings require not only excellent adhesive properties but also the ability to form *in situ* and adapt to wound shape. Li et al. developed a light-curable hydrogel that has the characteristics of “Transparency,” “Epithelium & Stroma generation,” as well as “Suturelessness” and “Toughness” (T.E.S.T) for repairing corneal trauma. Following 5 min of light irradiation, the hydrogels rapidly formed *in situ*. Importantly, multiple interfacial bonds enabled the hydrogel to firmly adhere to corneal tissue, and the multiple cross-links allowed the hydrogel to withstand nearly 600% deformation. (Li et al., 2023). Liu et al. synthesized a hybrid hydrogel by photo-crosslinking arginine-based poly (ester amide) (Arg-PEA) and hyaluronic acid (HA-MA). This hydrogel achieved *in situ* gelation and facilitated the healing of irregular wounds. In addition, it exhibited antioxidant, anti-inflammatory, fibrotic, and angiogenic effects during healing (Liu et al., 2022a).

In addition to movement, inflammation poses a major challenge to wound healing. Therefore, the control of inflammation is essential for tissue regeneration and repair after injury. Xu et al. demonstrated that a QMPD hydrogel composed of methacrylate anhydride (MA) grafted quaternary ammonium chitosan (QCS-MA), polyvinylpyrrolidone (PVP), and dopamine (DA) effectively decreased the inflammatory response in *Staphylococcus aureus*-infected rats. Moreover, it expedited wound healing when exposed to 808-nm NIR laser irradiation (Xu et al., 2023). Zhu et al. successfully fabricated a metformin-carrying CuPDA nanoparticle hybrid hydrogel (Met@CuPDA NPs/HG) that exhibited antibacterial, anti-inflammatory, and angiogenic properties under NIR irradiation. The exceptional photothermal responsiveness of the hydrogel was attributed to CuPDA NPs. Further, hematoxylin and eosin (H&E) staining and Masson’s trichrome staining revealed that the Met@CuPDA NPs/HG effectively reduced inflammation and accelerated diabetic wound healing (Zhu et al., 2023).

**FIGURE 9:**
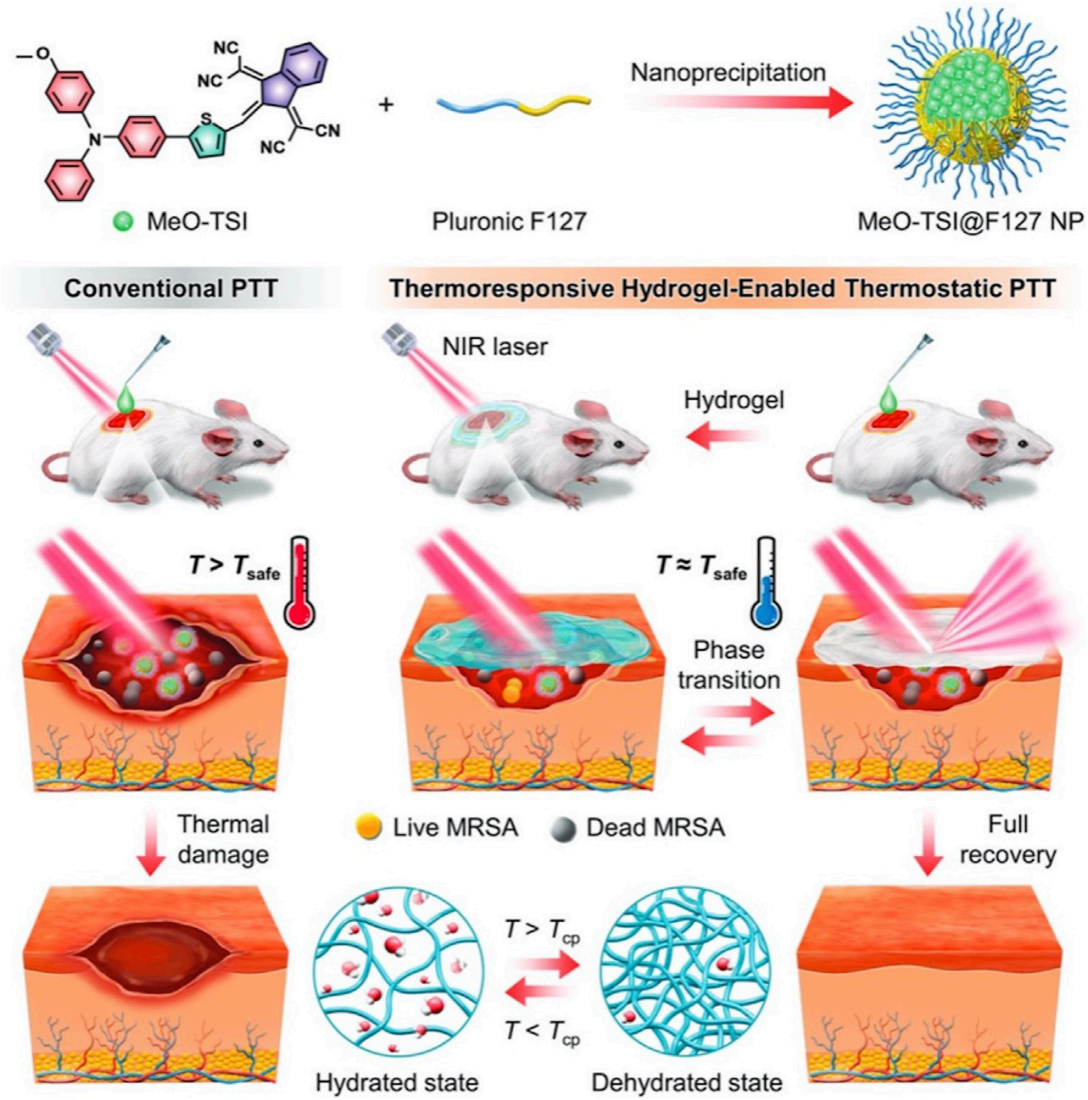
Schematic illustration showing the thermoresponsive hydrogel-enabled thermostatic PTT with negligible thermal damages for enhanced healing of bacteria-infected wounds. Reproduced from ref (Fu et al., 2023) with permission from Wiley, copyright 2023.

The healthy tissue surrounding lesions is also susceptible to damage at high temperatures (Shen et al., 2020). Therefore, the NIR-responsive hydrogels should achieve tissue regeneration within a safe temperature range. As such, Fu et al. developed a thermo-regulated PTT system using a thermo-responsive hydrogel. Under NIR irradiation, the ability of the P(NIPAM-AM) hydrogel to undergo phase transition can modulate the heating behavior of photothermal materials (Figure 9). This ensures the effective protection of healthy tissue and organs (Fu et al., 2023). Similarly, Sheng et al. developed a new kind of “hot spring effect” hydrogel, which was based on fayalite (FA) and *N,O*-carboxymethyl chitosan (NOCS) for wound healing. The composite hydrogel not only exhibited the good release of Fe^2+^/SiO_4_^4-^ ions, but also demonstrated excellent photothermal properties. In diabetic mouse models, the intensity of CD31 staining was highest in the FA-NOCS-L group after 7 days, indicating that neovascularization of diabetic wounds was significantly enhanced (Sheng et al., 2021).

The repair of damaged bones is intricate and involves the complex interplay between bone cells, extracellular components, and osteoinductive factors (Lin X. et al., 2020; Kosinski et al., 2020; Zhou B. et al., 2023; Yu et al., 2023). Therefore, the regeneration and repair of bone defects require an appropriate temperature. Wu et al. incorporated PDA into a GelMA hydrogel, and methyl methacrylate (MMA) was introduced to improve its mechanical performance. Under NIR irradiation, the hydrogel achieved a mild temperature of 40°C–42 °C, promoting bone regeneration (Wu et al., 2022). Similarly, Wu et al. developed an injectable photocurable hydrogel (AMAD/MP) using methacrylated alginate (Alg-MA), dopamine-grafted alginate (Alg-DA), and PDA-coated Ti_3_C_2_ MXene nanosheets (MXene@PDA NSs). Under NIR irradiation, the composite hydrogel achieved a mild temperature (42°C ± 0.5 °C) during PTT, resulting in enhanced osteogenic activity (Wu et al., 2023).

**FIGURE 10:**
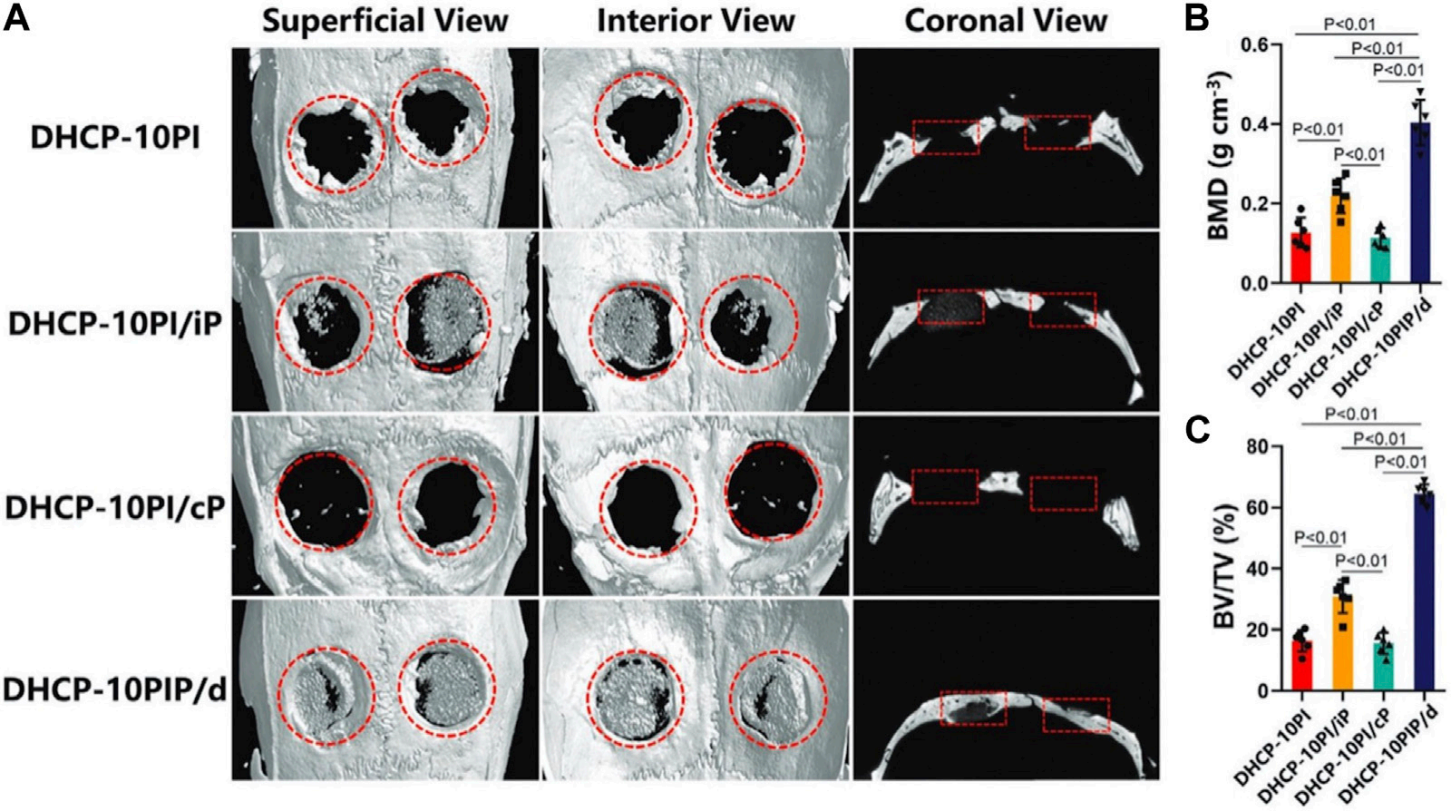
Micro-CT results of bone regeneration in osteoporosis rats at week 12. **(A)** 3D reconstruction images of the defect sites by micro-CT. **(B)** BMD (bone mineral density) and **(C)** BV/TV (bone volume/total volume) in each group. Reproduced from ref (Kuang et al., 2021) with permission from Wiley, copyright 2021.

As mentioned, photothermal hydrogels can use NIR irradiation to achieve controllable drug delivery and heal damaged tissue. For example, parathyroid hormone (PTH) has a strong effect on the balance between osteoblasts and osteoclasts (Rachner et al., 2019; Zhang and Song, 2020; Eastman et al., 2021). Therefore, Wang et al. developed an NIR-responsive reduced graphene oxide-loaded chitosan (CS/rGO) hydrogel designed for the pulsed release of teriparatide (PTH 1-34). This composite hydrogel demonstrated remarkable effectiveness in the treatment of osteoporotic bone regeneration (Wang X. et al., 2021). In addition, Kuang et al. created a DHCP-10PIP/d hydrogel loaded with PTH, achieving dual-mode (continuous + pulse mode) release under NIR irradiation. In the ovariectomized (OVX) rat model of cranial defect, the new bone regeneration in the DHCP-10PIP/d group (BMD: 0.404 ± 0.052; BV/TV: 64.405% ± 2.826%) surpassed that of other groups (Figure 10) (Kuang et al., 2021).

In addition, the regeneration of dental tissue is crucial for improving oral health after illness. Given the complex and irregular structure of dental tissue, the use of hydrogels for regeneration offers significant advantages (Tang et al., 2020). In one example, an injectable photocrosslinked hydrogel containing methacrylylated silk fibroin (RSFMA) and methacrylylated hyaluronic acid (MeHA) loaded with human dental pulp stem cells (hDPSCs) was designed to promote pulp regeneration and address the intricate and irregular anatomy of the root canal system (Wang et al., 2022a).

## 6 Conclusion and prospective

In this review, we have summarized the latest research progress in photothermal hydrogels for infection control and tissue regeneration. First, we introduced the main types of PTAs and their advances in photothermal antibacterial application. Then, the design principles of the photothermal hydrogels were discussed, and their advantages and disadvantages were compared. Second, we summarized the potential mechanisms of photothermal effects in antibacterial activity, angiogenesis, and tissue reconstruction. Finally, we concluded the applications of photothermal hydrogels in infection control and tissue regeneration.

Despite the encouraging achievements in the development of photothermal hydrogels for infection control and tissue regeneration, challenges and unresolved issues remain. Future research directions include the following: 1) Several photothermal hydrogels suffer rapid biodegradation and have insufficient stability in the long term, resulting in performance loss. To address this issue, it is crucial to adjust material formulations and refine processing techniques. For instance, the incorporation of more biodegradable biomaterials, optimization of the degree of cross-linking, and introduction of stability-enhancing agents could improve the biocompatibility, stability, and photothermal effects (Liang et al., 2019; Qi et al., 2021; Liu et al., 2022b). Of particular importance, the careful selection of suitable materials for specific applications is paramount. Resolving these issues is a prerequisite for advancing their clinical applications. 2) The photothermal effect has a limited tissue depth, particularly in tissues that scatter light strongly, preventing the treatment of deep-tissue infections. To overcome this issue, light wavelengths having better tissue penetration should be selected, for example, the NIR rather the UV regions (Wu and Butt, 2016). In addition, the introduction of materials such as NPs into tissues can enhance the light scattering, thereby increasing light propagation and the range of photothermal effects (Chen et al., 2019). Finally, achieving a balance between treatment efficacy and deep tissue penetration requires the careful adjustment of the intensity and duration of light irradiation. 3) Although some materials may have ideal performance characteristics, they can be costly or difficult to prepare, which reduces the feasibility of large-scale production and application. To achieve widespread use, production optimization, cost reduction, and material scalability are essential (Deng et al., 2020; Ni et al., 2022; Zhao et al., 2022).

In addition to these points, clinical trials are required to validate their safety and effectiveness and expedite the transformation of photothermal hydrogels from the laboratory to the clinic. Key to this, collaboration between multidisciplinary research teams is crucial to addressing the many complex challenges in the application of photothermal hydrogels for infection control and tissue regeneration.
